# Monoclonal Antibody Aggregation Associated with Free Radical Induced Oxidation

**DOI:** 10.3390/ijms22083952

**Published:** 2021-04-12

**Authors:** Kai Zheng, Diya Ren, Y. John Wang, Wayne Lilyestrom, Thomas Scherer, Justin K. Y. Hong, Junyan A. Ji

**Affiliations:** 1 Pharmaceutical Development, Genentech, South San Francisco, CA 94080, USA; pharmulator@yahoo.com (Y.J.W.); wayne.lilyestrom@gmail.com (W.L.); tms_508@yahoo.com (T.S.); jji@gene.com (J.A.J.); 2 Oceanside Pharmaceutical Technical Development, Genentech, Oceanside, CA 92056, USA; diya.ren@gmail.com; 3 Department of Pharmaceutical Sciences, University of Michigan, Ann Arbor, MI 48109, USA; hkyjustin@gmail.com

**Keywords:** monoclonal antibody, free radical, protein aggregation, oxidation, excipient

## Abstract

Oxidation is an important degradation pathway of protein drugs. The susceptibility to oxidation is a common concern for therapeutic proteins as it may impact product efficacy and patient safety. In this work, we used 2,2′-azobis (2-amidinopropane) dihydrochloride (AAPH) as an oxidative stress reagent to evaluate the oxidation of therapeutic antibodies. In addition to the oxidation of methionine (Met) and tryptophan (Trp) residues, we also observed an increase of protein aggregation. Size-exclusion chromatography and multi-angle light scattering showed that the soluble aggregates induced by AAPH consist of dimer, tetramer, and higher-order aggregate species. Sodium dodecyl sulfate polyacrylamide gel electrophoresis indicated that inter-molecular disulfide bonds contributed to the protein aggregation. Furthermore, intrinsic fluorescence spectra suggested that dimerization of tyrosine (Tyr) residues could account for the non-reducible cross-links. An excipient screening study demonstrated that Trp, pyridoxine, or Tyr could effectively reduce protein aggregation due to oxidative stress. This work provides valuable insight into the mechanisms of oxidative-stress induced protein aggregation, as well as strategies to minimize such aggregate formation during the development and storage of therapeutic proteins.

## 1. Introduction

Oxidation-induced degradation of therapeutic proteins is commonly observed during pharmaceutical manufacturing, handling, and storage [1,2,3]. These oxidation reactions occur when there are activated oxygen species including singlet oxygen (^1^O_2_), superoxide radical (O_2_•^−^), hydroxyl radical (•OH), and peroxide (-OO-) in the environment. Reactive oxygen species can be created through light, heat, free radicals, or transition metals [4,5]. These species can oxidize methionine (Met), tryptophan (Trp), histidine (His), tyrosine (Tyr), and cysteine (Cys) residues in proteins [6]. For example, it has been reported that ultraviolet (UV) irradiation induced oxidation of Met and Trp residues in a monoclonal antibody (mAb) [7], thermal or chemical stresses caused Met oxidation in proteins [8,9,10], and free radicals promoted Tyr oxidation in ATPase [11].

Oxidative modifications can alter protein’s secondary and tertiary structures [12], hydrophobicity [13], stability [14], biological activity [14], and plasma circulation half-life [15]. Owing to these potential impacts, oxidation is typically an important quality attribute to be closely monitored during the development of therapeutic proteins. Various stress models including light and chemical stresses are often used to identify potential oxidation hot spots in therapeutic proteins and to characterize the vulnerability of labile residues to oxidation. *Tert*-butyl hydroperoxide (*t*-BHP), hydrogen peroxide (H_2_O_2_), or 2,2′-azobis (2-amidinopropane) dihydrochloride (AAPH) are often used as chemical stress reagents to evaluate protein oxidation [6,8,9,10,12,16,17,18]. In general, *t*-BHP and H_2_O_2_ primarily oxidize Met residues via nucleophilic substitution reactions. In contrast, AAPH (a water-soluble radical initiator) oxidizes both Met and Trp residues [8,19] through free radical reactions. The azo compound, AAPH, is thermally unstable and can generate alkyl radicals at elevated temperatures. In the presence of oxygen, alkyl radicals can form peroxyl radicals [20], which can further form alkoxyl radicals [11]. These radical species can effectively oxidize Met, Trp, and other oxidation-labile residues in proteins. One advantage of using an azo-radical initiator, such as AAPH or azobisisobutyronitrile, as an oxidative reagent is that it can produce a controllable and reproducible amount of oxidizing species [19,21,22].

In previous work conducted by Ji et al. [9], researchers used oxidative reagents of *t*-BHP, H_2_O_2_, and AAPH to study the mechanism of Met, Trp, and His oxidation in parathyroid hormone. Although Ji et al.’s work provided valuable insights into the oxidation mechanisms and the corresponding stabilization strategy, the model protein, parathyroid hormone, used in the study is a small protein with minimal tertiary structure. On the other hand, oxidation in large proteins, such as mAbs, may not only cause oxidation of Met, Trp, His, or other labile residues, but also induce additional physicochemical degradations. For example, the previous work did not assess aggregation that can be observed during protein oxidation. Therefore, in this work, we evaluated the oxidative degradation of therapeutic mAbs under the AAPH stress, where we observed both mAb oxidation and aggregation. Protein aggregation is often induced by physical stresses, such as agitation [23], freeze/thawing, and freeze/drying processes [24]. The AAPH-induced aggregation observed here is likely formed through covalent bonds as a result of free radical reactions. Protein aggregates can potentially trigger immune responses and are considered as a critical quality attribute for therapeutic proteins [25]. Thus, it is important to have a good understanding of the formation and the nature of protein aggregates associated with protein oxidation. In addition, this study also provides insight to reduce protein aggregation when exposing to oxidative reagents during therapeutic protein production and storage.

## 2. Results

### 2.1. Aggregate Formation under the AAPH Stress

**Figure 1:**
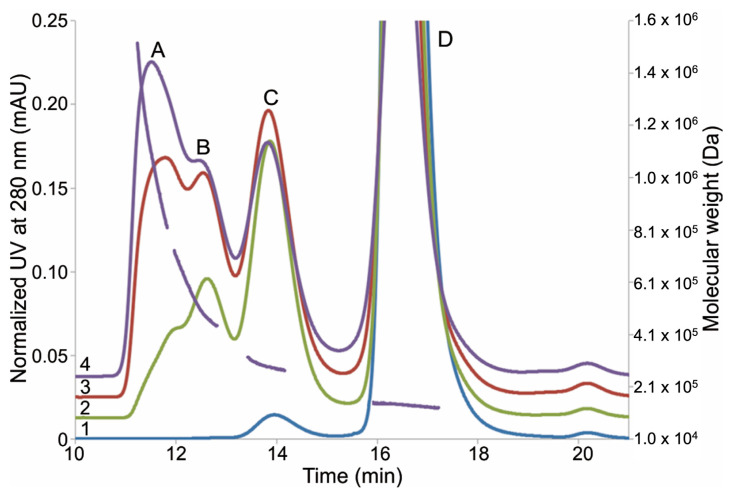
Expanded view of mAb1 size exclusion chromatography (SEC) profiles after 2,2′-azobis (2-amidinopropane) dihydrochloride (AAPH) stress. Chromatogram 1: control sample (mAb1 without the AAPH stress); chromatogram 2: mAb1 oxidized by 1 mM AAPH; chromatogram 3: mAb1 oxidized by 3 mM AAPH; 4: mAb1 oxidized by 5 mM AAPH. Molecular weights of peaks (A–D) for mAb1 oxidized by 5 mM AAPH are calculated and overlaid in the figure as the dashed line.

AAPH was used to assess the oxidative degradation of mAbs during the formulation development. Size exclusion chromatography (SEC) of the oxidized mAb1 revealed a substantial increase of protein aggregates (SEC; Figure 1). The percentages of aggregates, monomer, and fragments were calculated based on SEC peak areas and are summarized in Table 1. Increasing AAPH concentrations results in a quantitative loss of monomer with corresponding increases of aggregates (SEC peaks A-C between 11 and 15 min in Figure 1). The impact on mAb1 fragmentation under the oxidative stress is relatively subtle. SEC coupled with multi-angle light scattering (SEC-MALS) was used to further characterize the aggregates in the mAb1 sample stressed with 5 mM AAPH. SEC-MALS data indicated that the aggregates upon the oxidative stress contained dimer (peak C, 3.0 × 10^5^ Da) and higher-order aggregates (peaks A/B 5.2 × 10^5^–1.4 × 10^6^ Da) (Figure 1).

### 2.2. Fraction Collection from SEC

**Figure 2:**
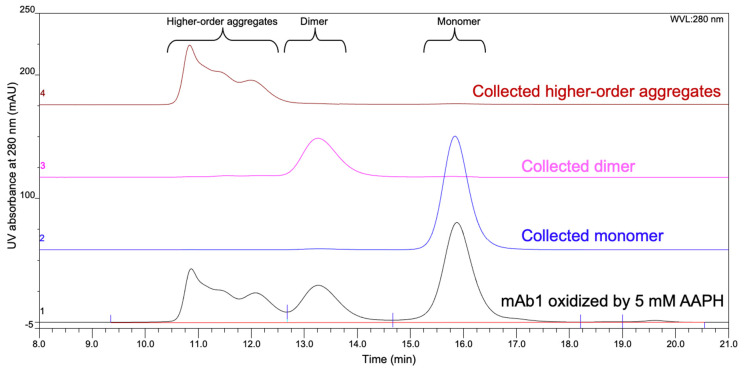
SEC of the collected fractions of mAb1 oxidized by 5 mM AAPH. Chromatogram 1: before fraction collection; chromatogram 2: re-injected monomer fraction; chromatogram 3: re-injected dimer fraction; chromatogram 4: re-injected higher-order aggregates fraction.

Given that mAb1 stressed by 5 mM AAPH showed the most pronounced changes, we henceforth collected fractions of 5 mM AAPH stressed mAb1 using SEC for further characterization. Higher-order aggregates (peaks A and B in Figure 1), dimer (peak C in Figure 1), and monomer (peak D in Figure 1) peaks were collected and buffer exchanged to 20 mM sodium acetate buffer at pH 5.5. The sizes and purity of the enriched species were further confirmed by the SEC analysis. Each sample eluted as a single peak in SEC (Figure 2). The purity of higher-order aggregates, dimer, and monomer was estimated to be 99.1%, 93.2%, and 98.4%, respectively, based on SEC peak areas.

### 2.3. Aggregate Characterization

**Figure 3:**
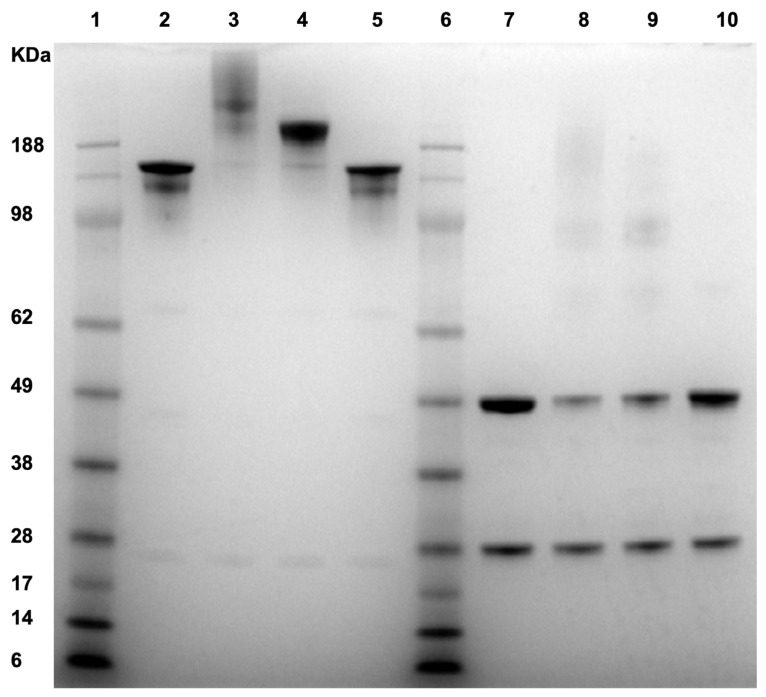
Sodium dodecyl sulfate polyacrylamide gel electrophoresis (SDS-PAGE) of the collected fractions from SEC. Lane 1: protein standards; lane 2: non-reduced control sample (no AAPH stressed mAb1); lane 3: non-reduced higher-order aggregates; lane 4: non-reduced dimer; lane 5: non-reduced monomer; lane 6: protein standards; lane 7: reduced control sample (no AAPH stressed mAb1); lane 8: reduced higher-order aggregates; lane 9: reduced dimer; lane 10: reduced monomer.

Sodium dodecyl sulfate polyacrylamide gel electrophoresis (SDS-PAGE) analysis with Coomassie blue staining was used to characterize properties of the fractions collected from SEC. Both reduced and non-reduced samples were analyzed by SDS-PAGE (Figure 3). Under the non-reduced condition, the un-oxidized control (lane 2 in the SDS-PAGE gel) runs with a molecular weight (MW) of ~150 kDa, as expected. Higher-order aggregates (lane 3 in the gel) showed multiple bands with MWs higher than the 188 kDa standard, indicating that higher-order aggregates consist of covalently linked multimers that do not dissociate under the denaturing condition. The dimer species (lane 4 in the gel) showed a single band with a MW higher than 188 kDa, indicating that the dimer is also covalently linked. The monomer peak (lane 5 in the gel) has a MW similar to the un-oxidized control sample in lane 2. Under the reduced condition, both the un-oxidized control sample and the monomer fraction (lanes 7 and 10 in the gel, respectively) dissociated into light chains (~25 kDa) and heavy chains (~50 kDa). Reduced higher-order aggregates (lane 8 in the gel) dissociated to light chains, heavy chains, and a few faint bands with MWs of 100 kDa or higher. These results indicate that inter-molecular disulfide cross-links contribute to the formation of higher-order aggregates. The aggregates with MWs of 100 kDa or higher suggest that the aggregates also contain non-reducible inter-molecular cross-links. The mass of 100 kDa suggests that the cross-links may be formed between two heavy chains. The dimer species (lane 9 in the gel) dissociated mainly to light chains and heavy chains upon reduction, indicating that inter-molecular disulfide bonds played a major role in mAb1 dimerization. In addition, a few faint bands with MWs of 100 kDa or higher were observed as well, suggesting other inter-molecular cross-links also contributed to dimerization.

### 2.4. Mass Spectrometry with Trypsin Digestion

**Figure 4:**
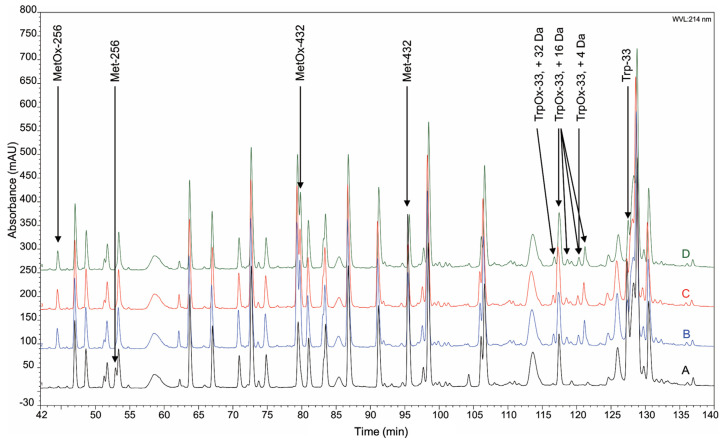
Expanded view of tryptic maps of collected SEC fractions of mAb1. Chromatogram A: control sample (no AAPH stressed mAb1); chromatogram B: collected monomer fraction from SEC; chromatogram C: collected dimer fraction from SEC; chromatogram D: collected higher-order aggregate fraction from SEC. Oxidized Met and Trp products and their parent peaks are labeled in the figure.

Because the aggregates contain non-reducible cross-links, we used the tryptic peptide maps to characterize chemical modifications of mAb1 after the AAPH stress (Figure 4). MAb1 contains Trp-33 in the CDR and Met-256 and Met-432 in the Fc region. Oxidation of these residues in higher-order aggregates, dimer, and monomer samples was observed as expected (indicated by arrows in Figure 4). Upon oxidation, the hydrophobicity of oxidized Met and Trp residues decreased and, therefore, eluted early on the reverse phase high-performance liquid chromatography (HPLC). Oxidation of Met-256 and Met-432 resulted in a single peak of MetOx-256 and MetOx-432 on the chromatograms, respectively. On the other hand, oxidation of Trp is more complicated than oxidation of Met because the indole side-chain of Trp could undergo various reactions during oxidation and lead to products with mass increases of +4, +16, +20, and +32 [26]; indeed, multiple peaks representing various oxidation products of Trp-33 were observed (Figure 4). These Trp-33 oxidation products eluted at 116–122 min. Some Trp oxidation products were visible as new peaks in the chromatograms and others co-eluted with other peptides. The mass increase of each Trp oxidation product is labeled in Figure 4. Except Met and Trp oxidation, we did not observe any other new peaks in the chromatogram of the higher-order aggregate or dimer fraction that indicated non-reducible cross-links, as suggested by the data of SDS-PAGE. A reasonable explanation could be that the tryptic peptide containing cross-linking sites is very large, which has low ionization efficiency and is difficult to be detected by mass spectrometry. Another possible reason is that there are multiple cross-linking sites with low abundance, which can be detected by SDS-PAGE as broad faint bands, but not by mass spectrometry once eluted at different regions on the chromatogram.

### 2.5. Intrinsic Fluorescence

**Figure 5:**
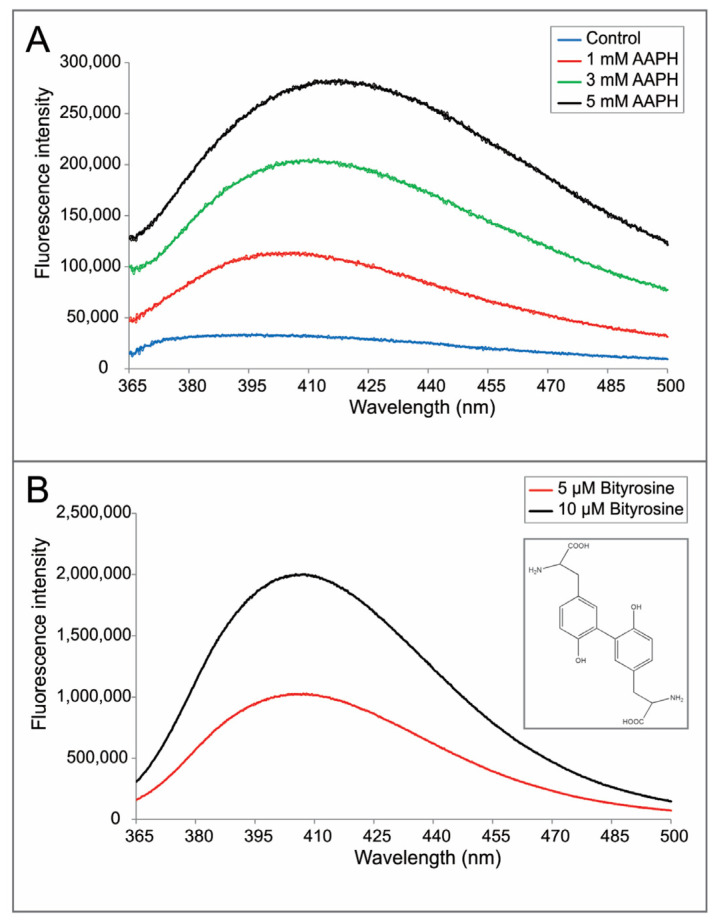
(**A**) Intrinsic fluorescence spectra of the control sample (no AAPH stressed mAb1) and mAb1 after stressing by 1, 3, and 5 mM AAPH. (**B**) Intrinsic fluorescence spectra of bityrosine standard at 5 µM and 10 µM. Inset: chemical structure of bityrosine.

Given that mass spectrometry cannot identify non-reducible cross-linking sites, we used intrinsic fluorescence spectroscopy for further characterization. Fluorescence emission spectra of AAPH-oxidized samples under the excitation wavelength of 325 nm are shown in Figure 5A. The broad emission peak between 400 and 430 nm suggests the existence of bityrosine cross-links after AAPH oxidation, as described in a previous report [11]. This result indicated that cross-links between Tyr residues could account for the non-reducible bands in SDS-PAGE (lanes 8 and 9 in Figure 3). In addition, we collected emission spectra of bityrosine standard to further support this hypothesis (Figure 5B). Spectra of the standard and oxidized mAb1 exhibited similar fluorescent peaks between 400 and 430 nm. Furthermore, a higher intensity of fluorescent emission was observed with increased AAPH concentration (Figure 5A), indicating an increasing trend of bityrosine cross-links with higher levels of AAPH.

### 2.6. Aggregation Inhibition

**Figure 6:**
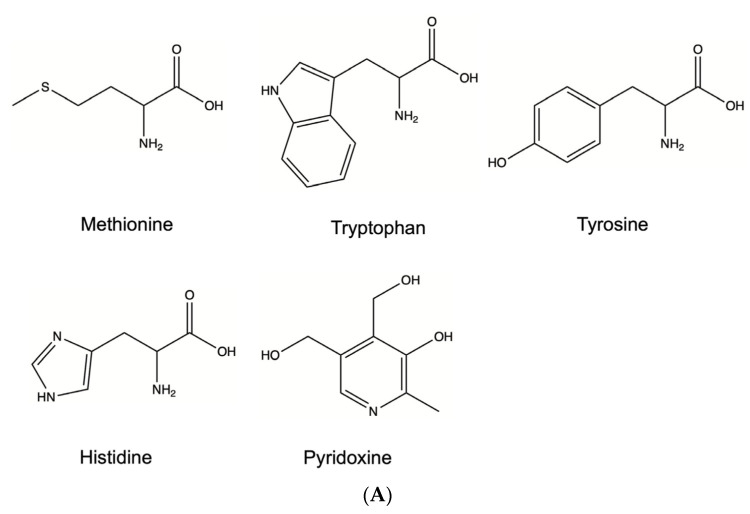

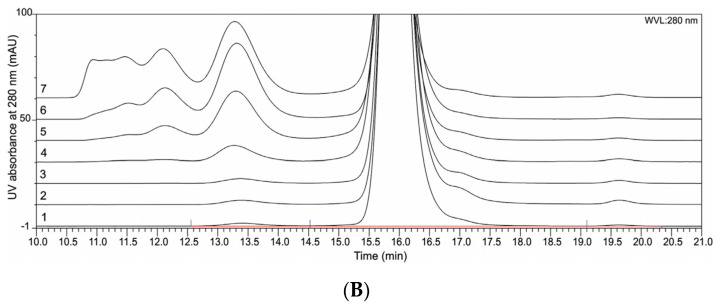
(**A**) Structure of tested aggregation inhibitors. (**B**) Expanded view of mAb1 SEC profiles for excipient screening (except the negative control, all samples contained 1 mM AAPH). Chromatogram 1: negative control sample (no AAPH stressed mAb1); chromatogram 2: sample contained 2 mM Trp; chromatogram 3: sample contained 2 mM pyridoxine; chromatogram 4: sample contained 2 mM Tyr; chromatogram 5: sample contained 2 mM His; chromatogram 6: sample contained 2 mM Met; chromatogram 7: positive control sample (AAPH stressed mAb1). All samples contained 10 mg/mL mAb1 and were incubated at 40 °C for 72 h prior to SEC analysis.

Because protein aggregates could induce immune responses and impact patient safety, it is important to protect therapeutic proteins from aggregation, especially when free radicals may be present in the protein solution [27]. In this study, we first screened Met, Trp, Tyr, His, and pyridoxine as aggregation inhibitors for the following reasons: free Met or Trp was added because Met and Trp oxidation was observed in mAb1 under the AAPH stress; free Tyr was evaluated as the fluorescence data suggest the formation of bityrosine cross-links; free His was selected because His oxidation has been reported in other studies [9,28]; and pyridoxine was included as a free radical scavenger [29]. We did not include ascorbic acid and N-acetyl cysteine (NAC), which are typical anti-oxidants, in the screening study as they will not be suitable excipients for therapeutic proteins: ascorbic acid has been shown to cause undesirable reactions with proteins and NAC may cause disulfide exchange [9]. The structures of the screened inhibitors are shown in Figure 6A. After incubation at 40 °C for 72 h, the aggregate levels were evaluated using SEC. The chromatograms shown in Figure 6B indicated that Trp, pyridoxine, and Tyr can effectively protect protein from oxidation-induced aggregation under the AAPH stress. His and Met also decreased protein aggregation, but not to the same extent as Trp, pyridoxine, and Tyr.

**Figure 7:**
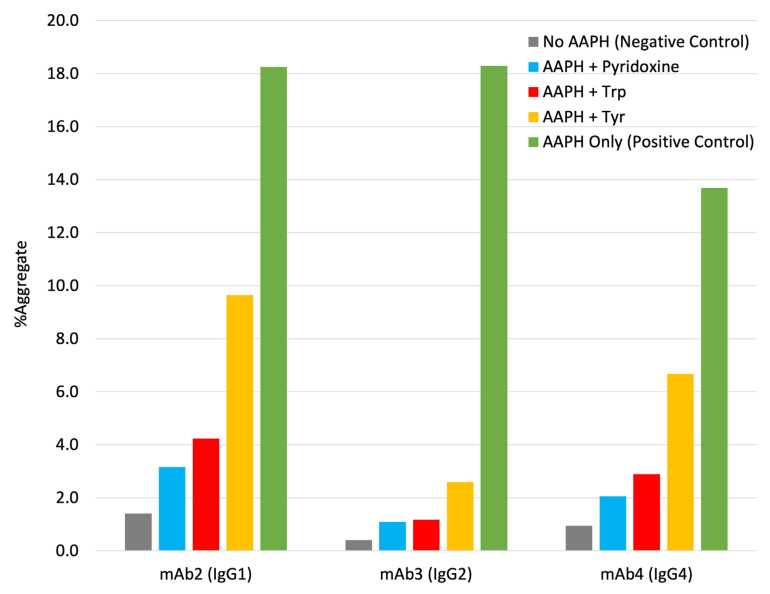
Aggregation of various mAbs under 1 mM AAPH stress after 72 h at 40 °C. Grey bar: no AAPH stressed mAb (negative control); light blue bar: AAPH stressed mAb containing pyridoxine; red bar: AAPH stressed mAb containing Trp; yellow bar: AAPH stressed mAb containing Tyr; green bar: AAPH stressed mAb without aggregation inhibitors (positive control).

With this observation, we further expanded the assessment of the aggregation inhibition effect of Trp, pyridoxine, and Tyr on other types of mAbs, including another IgG1 mAb (mAb2), an IgG2 mAb (mAb3), and an IgG4 mAb (mAb4). Under the AAPH stress, all three mAbs showed substantial increases of aggregates (~12–18%) after 72 h of storage at 40 °C (Figure 7), while the addition of Trp, pyridoxine, or Tyr can significantly reduce the aggregation, with the order of pyridoxine ≥ Trp > Tyr.

## 3. Discussion

Protein oxidation is a very complicated process that could be triggered by chemical [4,6,30,31,32,33] or light [34,35,36] stresses and produces a diverse collection of oxidation products. In this study, we used AAPH as a chemical stress reagent to characterize oxidation of therapeutic mAbs. In addition to expected Met and Trp oxidation, we observed substantial protein aggregation. Although aggregated species of therapeutic proteins could cause immunogenicity risks and impact patient safety, there are few reports having a thorough investigation on oxidative-stress induced protein aggregation. Our work revealed that the newly formed aggregates are primarily covalently linked. Our results also differ from some previous reports. One report described a study that used *t*-BHP to assess Trp oxidation in a recombinant IgG1 mAb [17]. In that study, only a small increase in aggregation was observed by SEC, while our data showed a remarkable increase in aggregation. In another study [11], AAPH was used to oxidize Ca^2+^-ATPase. There, protein aggregation was observed, but the contribution from disulfide cross-links was ruled out, whereas the data in our study suggested that a majority of aggregates in mAbs were covalently linked through inter-molecular disulfide bonds under the AAPH stress. Because an mAb contains a large number of disulfide bridges, inter-molecular disulfide cross-links could begin with the reduction of a disulfide bridge by free radicals and then the formation of a cysteine thiyl radical intermediate, which is not stable and can react with another disulfide bridge in an adjacent molecule to form an inter-molecular disulfide cross-link [37].

**Figure 8:**
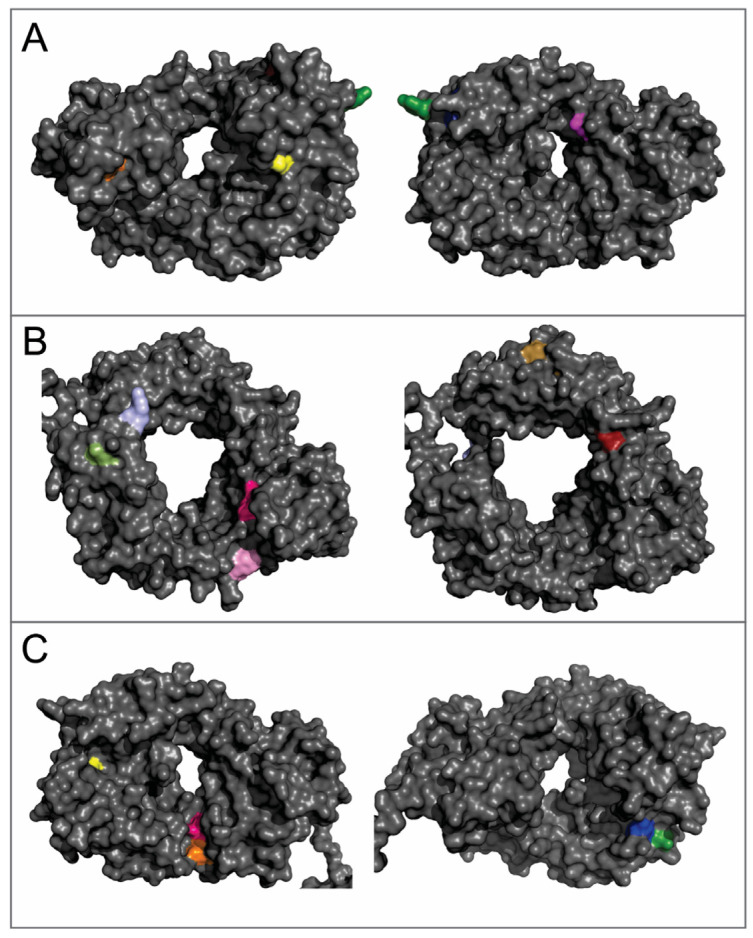
Homology models of mAb1 with the location of Tyr residues. (**A**) The heavy chain Fab region; (**B**) the heavy chain Fc region; and (**C**) the light chain. All Tyr residues are in color; the other residues are grey.

In addition to disulfide cross-links, bityrosine cross-links can also contribute to the covalent aggregates in mAbs. Based on a number of previous reports [11,38,39], bityrosine cross-links may be formed through a phenoxyl radical intermediate, which is the product of the reaction between free radicals and tyrosine side-chains. Two phenoxyl radicals then recombine to form an inter-molecular or intra-molecular cross-link between two benzyl-ring side chains of Tyr residues. In this work, we observed fluorescence emission spectra that are characteristic of bityrosine, which suggests the formation of bityrosine cross-links under the AAPH stress (Figure 5A). As mAbs possess complex structures and numerous Tyr residues, it increases the challenge to identify the specific bityrosine cross-linking site compared with small proteins. For mAb1, in this study, there are eighteen Tyr residues in the heavy chain and ten Tyr residues in the light chain. The observed fluorescent peak in oxidized samples can be contributed from a single bityrosine cross-linking site or multiple cross-linking sites. To further characterize bityrosine cross-links, we generated homology models of heavy chain Fab, heavy chain Fc, and light chain regions (Figure 8A–C). In the heavy chain Fab region, only the side chain of Tyr-57 is fully exposed to solvent (the green color residue in Figure 8A). In heavy chain Fc and light chain regions (Figure 8B,C), all Tyr residues are buried inside. Thus, it is likely that the non-reducible inter-molecular cross-links are formed through heavy chain Fab regions. If that is the case, the G^41^……DY^57^A…K^62^ peptide that dimerized through Tyr-57 should be observed in tryptic peptide mapping analysis (Figure 4). However, after careful analysis of mass spectroscopy data, such dimer peptide was not identified. This result may be because the dimer peptide is too hydrophobic to elute. In addition, as mentioned previously, the dimer peptide may lack sufficient ionization efficiency. Furthermore, during the AAPH stress, the mAb tertiary structure could be altered, and other Tyr residues may be exposed and cross-linked. In that case, protein aggregates contain heterogeneous bityrosine cross-links and the abundance of tryptic digested peptides that contain a specific cross-link would be too low to be detected by mass spectroscopy. A possible approach to identify cross-linking sites is to synthesize some peptides containing bityrosine cross-links and use them as standard compounds for peptide mapping analysis. Another strategy is to use site-specific mutations to investigate whether specific residues, such as Tyr-57 in the heavy chain, are responsible for the formation of inter-molecular cross-links. In addition, in-gel digestion may be another approach to identify cross-linking sites by combining SDS-PAGE and mass spectroscopy analysis. These alternative approaches are worthy of further investigation and can be explored in a separate study.

In this work, we assessed Trp, pyridoxine, and Tyr as aggregation inhibitors for IgG1, IgG2, and IgG4 types of mAbs, which covers a good range of antibody platforms as potential therapeutic proteins. To our knowledge, this is the first report to assess IgG1, IgG2, and IgG4 mAbs side-by-side for these inhibitors. Our results showed that these inhibitors can effectively reduce protein aggregation induced by free radicals for the tested mAbs. In general, these inhibitors protect proteins by consuming free radicals in the solution. Therefore, they can also provide a certain level of protection to oxidation labile residues in proteins, which was demonstrated in the previous report [9]. Ji et al.’s work showed that free Trp or pyridoxine in the formulation can effectively protect Trp oxidation in parathyroid hormone under the AAPH stress, while Tyr exhibited only slight protection of the Trp residue. Thus, to protect against both aggregation and oxidation of therapeutic proteins, we can consider combining the inhibitors tested in this work, or their homologs [40], with other anti-oxidant excipients in protein formulations. This strategy can be an effective approach, especially for large proteins that can have complex degradation mechanisms under oxidative stresses. Lastly, besides protection effectiveness, researchers also need to assess whether these excipients form adducts to therapeutic proteins and the impact of the oxidative end-products of these excipients. Though these experiments had not been done in this work because they were outside the scope of the intended study, they should be considered during formulation development when using these excipients.

## 4. Materials and Methods

### 4.1. Reagents

The reagents used in this study included AAPH (EMD Chemicals, Gibbstown, NJ, USA), tris (2-carboxyethyl) phosphine (TCEP), trifluoroacetic acid (TFA), formic acid (Thermo Scientific, Waltham, MA, USA), DL-dithiothreitol (DTT), iodoacetic acid (IAA), L-Met, L-Trp, L-Tyr, pyridoxine (vitamin B6) (Sigma-Aldrich, St. Louis, MO, USA), trypsin (Roche, Indianapolis, IN, USA), water, acetonitrile, potassium phosphate monobasic, potassium phosphate dibasic (J.T. Baker, Phillipsburg, NJ, USA), and potassium chloride (Mallinckrodt Chemicals, Phillipsburg, NJ, USA).

### 4.2. Protein Production

MAb1 and mAb2 are humanized mAbs based on a human IgG1 framework containing heavy chain V_H_III and light chain V_K_I subgroup sequences. MAb3 is a humanized mAb based on a human IgG2 framework containing heavy chain V_HI__β_ and light chain V_LKI_ subgroup sequences. MAb4 is a humanized mAb based on a human IgG4 framework. All mAbs are approximately 150 kDa in this study and were expressed in Chinese Hamster Ovary cells and purified through standard antibody purification procedures, including affinity chromatography, cation-exchange chromatography, anion-exchange chromatography, and final ultrafiltration and diafiltration steps. The purified mAbs thus obtained were further buffer exchanged into 20 mM sodium acetate buffer at pH 5.5 using 10,000 molecular weight cutoff (MWCO) Amicon Ultra-15 centrifugal filter devices (Millipore) and diluted to 20 mg/mL as the starting materials for the following studies in this work.

### 4.3. AAPH Stress of MAb1

Two milliliters of mAb1 at 10 mg/mL in 20 mM sodium acetate buffer at pH 5.5 was incubated with 1, 3, or 5 mM AAPH (final concentrations) for 24 h at 40 °C in 3 cc glass vials. The incubation was performed in chambers without illumination (in the dark). After incubation, all small molecule materials, including untreated AAPH, degraded AAPH, and other oxidants, were immediately removed from the antibody solution through buffer exchange using Zeba Spin desalting columns (Thermo Scientific), and the antibody was stored in 20 mM sodium acetate buffer at pH 5.5.

### 4.4. SEC Analysis

SEC provides quantitative information about the molecular size distribution of the protein. Size variants of the mAbs were separated using a TosoHaas TSK G3000SWXL column (7.8 × 300 mm) eluted isocratically with a mobile phase consisting of potassium phosphate and potassium chloride (pH 6.2). The separation was conducted at 25 °C with a flow rate of 0.5 mL/min. The detection wavelength was set at 280 nm.

Fractions of the entire peak of higher-order aggregate, dimer, and monomer species of mAb1 stressed by 5 mM AAPH were collected using the same column and chromatography conditions as mentioned above. The amount of mAb1 injected for fractionation was 0.5 mg. All collected fractions from SEC were concentrated to 10 mg/mL and buffer exchanged to 20 mM sodium acetate buffer at pH 5.5 using 10,000 MWCO Amicon Ultra-15 centrifugal filter devices.

### 4.5. SEC-MALS Analysis

An 18-angle Dawn enhanced optical system (EOS) light scattering detector with a 30 mW solid-state laser (λ = 690 nm) from Wyatt Technology was used for all SEC-MALS measurements. The sample temperature was maintained at 25 °C by a water-cooled Peltier temperature controller. The instrument was calibrated with 99.9% toluene (chromatography grade). For the SEC-MALS analysis, a detector gain setting of 100× was used for all photodiodes at fixed angles from 38° to 148°. Because the radius of gyration of mAb1 is <10 nm, 20 µL of a dilute solution (4 mg/mL) of mAb1 was used to normalize the voltage of the photodiodes relative to the 90° detector using a photodiode detector gain setting of 100× at the end of each experiment. During the experimental procedure, 10 µL of protein solution at 6 mg/mL was injected for each sample. Astra 5.3.4.20 Software (Wyatt Technology Corporation, Santa Barbara, CA, USA) was used to acquire and process the static light scattering data, with a dn/dc value of 0.185 mL/g applied to calculations with the appropriate extinction coefficient at UV 280 nm.

### 4.6. SDS-PAGE Analysis

The enriched monomer, dimer, and higher-order aggregates, along with an un-oxidized control, were denatured in the presence or absence of a reducing agent, TCEP, and analyzed by SDS-PAGE. Non-reduced samples were denatured by heating in the SDS-PAGE sample buffer at 60 °C for 10 min, while reduced samples were heated in the same buffer with the presence of TCEP. Each sample (3 µg) was loaded and separated on a 4–12% polyacrylamide gradient gel along with MW standards. The protein components were then visualized by Coomassie R-250 staining solution. The polyacrylamide gel, protein ladder, SDS-PAGE sample buffer, SDS-PAGE running buffer, Coomassie R-250 staining solution, and distaining solution were purchased from Invitrogen.

### 4.7. Tryptic Peptide Map

Control, higher-order aggregates, dimer, and monomer species were diluted to 0.5 mg/mL in 360 mM Tris, 2 mM EDTA, 40 mM DTT, 6 M guanidine hydrochloride buffer at pH 8.6. The diluted solution was incubated at 37 °C for 1 h to reduce all inter- and intra-chain disulfide bonds in mAb1. After the reduction, free thiol groups were protected through alkylation by adding freshly prepared IAA solution with a final IAA concentration of 90 mM. The alkylation reaction was completed in 15 min at room temperature with protection from light. The excess IAA was quenched by the addition of 1 M DTT solution to the final concentration of 50 mM. Immediately following the reduction and alkylation, samples were buffer exchanged to 25 mM Tris, 2 mM CaCl_2_ buffer at pH 8.2 using Zeba Spin desalting columns. Trypsin was then added at a ratio of 1:45 (w:w) to the antibody solution, which was subsequently incubated at 37 °C for 5 h. After digestion was complete, 10% TFA was added to a final concentration of 0.3% to quench trypsin activities. Tryptic peptides were injected into an Agilent 1200 HPLC system for liquid chromatography (LC) separation and a Thermo Fisher Scientific LTQ Orbitrap XL mass spectrometer for mass analysis.

Details of the LC separation method were reported previously [41]. In brief, gradient elution was applied to a Jupiter C18 column (2.0 × 250 mm, 5 µm, 300 Å) from Phenomenex. The flow rate was 0.25 mL/min and the column temperature was maintained at 55 °C. The injection amount was 35 µg and the detection wavelength was set to 214 nm. The mass spectrometry data of LC eluents were collected by an LTQ Orbitrap XL mass spectrometer and analyzed using Xcalibur 2.0.7 (Thermo Fisher Scientific) [42].

### 4.8. Intrinsic Fluorescence Spectroscopy

Intrinsic fluorescence spectra of mAb1 were collected using a Horiba Jobin Yvon Fluoromax-4 spectrofluorometer with a temperature-controlled water bath. All protein solutions contained 0.3 mg/mL mAb1 in 20 mM sodium acetate buffer at pH 5.5. The emission spectrum was collected from 365 to 500 nm with the excitation wavelength of 325 nm at 25 °C. A blank spectrum of the formulation buffer was subtracted from each sample spectrum. The excitation and emission slit widths were both set at 5 nm and data were collected with 0.2 nm increments and 0.2 s integration time.

The bityrosine standard was synthesized by Hande Science. The purity of bityrosine standard was >93%, as determined by the HPLC analysis. The emission spectra of 5 and 10 µM bityrosine standard were collected at the same condition as the mAb1 samples.

### 4.9. Aggregation Inhibitor Screening

In the initial screening, MAb1 at 10 mg/mL in 20 mM sodium acetate buffer at pH 5.5 with 1 mM AAPH was incubated with 2 mM aggregation inhibitors (Met, Trp, Tyr, His, or pyridoxine) at 40 °C for 72 h. In the subsequent screening, mAb2, mAb3, and mAb4 at 10 mg/mL in the same buffer including 1 mM AAPH were incubated with 2 mM aggregation inhibitors (Trp, pyridoxine, or Tyr) at 40 °C for 72 h. In addition, mAbs in 20 mM sodium acetate buffer at pH 5.5 without or with 1 mM AAPH were also incubated at 40 °C for 72 h as negative and positive controls, respectively, in both screening studies. After incubation, AAPH and other excipients were immediately removed from mAb solutions using Zeba Spin desalting columns. The purified samples were in 20 mM sodium acetate buffer at pH 5.5 and injected into a SEC column for size analysis.

## 5. Conclusions

In summary, the study results showed that free radicals can cause substantial mAb oxidation and aggregation. Free radicals can be introduced into therapeutic protein formulations from light exposure, excipient raw materials, and so on. Trp, pyridoxine, Tyr, or its homologs, in combination with other anti-oxidant excipients, can be an effective approach to protect therapeutic proteins against both aggregation and oxidation under oxidative stresses.

## Figures and Tables

**Table 1:** Size distribution of 2,2′-azobis (2-amidinopropane) dihydrochloride (AAPH)-stressed mAb1.

Sample	Aggregates (%)	Monomer (%)	Fragments (%)
AAPH Control	1.0	98.8	0.2
1 mM AAPH	33.7	65.9	0.4
3 mM AAPH	50.5	48.9	0.6
5 mM AAPH	55.8	43.5	0.7

MAb1 was incubated with AAPH for 24 h at 40 °C. Percentages were calculated from size exclusion chromatography (SEC) data.

## Abbreviations

AAPH, 2,2′-azobis (2-amidinopropane) dihydrochloride; Cys, cysteine; EOS, enhanced optical system; HIS, histidine; DTT, DL-dithiothreitol; HPLC, high-performance liquid chromatography; IAA, iodoacetic acid; LC, liquid chromatography; mAb, monoclonal antibody; Met, methionine; MW, molecular weight; MWCO, molecular weight cutoff; NAC, N-acetyl cysteine; SDS-PAGE, sodium dodecyl sulfate polyacrylamide gel electrophoresis; SEC, size-exclusion chromatography; SEC-MALS, size-exclusion chromatography and multi-angle light scattering; *t*-BHP, *tert*-butyl hydroperoxide; TCEP, tris(2-carboxyethyl) phosphine; Trp, tryptophan; Tyr, tyrosine; UV, ultraviolet.

## References

[1] Roy S., Mason B.D., Schöneich C.S., Carpenter J.F., Boone T.C., Kerwin B.A. (2009). Light-induced aggregation of type I soluble tumor necrosis factor receptor. J. Pharm. Sci..

[2] Ha E., Wang W., Wang Y.J. (2002). Peroxide formation in polysorbate 80 and protein stability. J. Pharm. Sci..

[3] Kroon D.J., Baldwin-Ferro A., Lalan P. (1992). Identification of sites of degradation in a therapeutic monoclonal antibody by peptide mapping. Pharm. Res..

[4] Hovorka S., Schöneich C. (2001). Oxidative degradation of pharmaceuticals: Theory, mechanisms and inhibition. J. Pharm. Sci..

[5] Wecksler A.T., Yin J., Tao P.L., Kabakoff B., Sreedhara A., Deperalta G. (2018). Photodisruption of the structurally conserved Cys-Cys-Trp triads leads to reduction-resistant scrambled intrachain disulfides in an IgG1 monoclonal antibody. Mol. Pharm..

[6] Li S., Schöneich C., Borchardt R.T. (1995). Chemical instability of protein pharmaceuticals: Mechanisms of oxidation and strategies for stabilization. Biotechnol. Bioeng..

[7] Wei Z., Feng J., Lin H.-Y., Mullapudi S., Bishop E., Tous G.I., Casas-Finet J., Hakki F., Strouse R., Schenerman M.A. (2007). Identification of a single tryptophan residue as critical for binding activity in a humanized monoclonal antibody against respiratory syncytial virus. Anal. Chem..

[8] Chumsae C., Gaza-Bulseco G., Sun J., Liu H. (2007). Comparison of methionine oxidation in thermal stability and chemically stressed samples of a fully human monoclonal antibody. J. Chromatogr. B.

[9] Ji J.A., Zhang B., Cheng W., Wang Y.J. (2009). Methionine, tryptophan, and histidine oxidation in a model protein, PTH: Mechanisms and stabilization. J. Pharm. Sci..

[10] Keck R.G. (1996). The use of t-butyl hydroperoxide as a probe for methionine oxidation in proteins. Anal. Biochem..

[11] Viner R.I., Krainev A.G., Williams T.D., Schöneich C., Bigelow D.J. (1997). Identification of oxidation-sensitive peptides within the cytoplasmic domain of the sarcoplasmic reticulum Ca^2+^-ATPase. Biochemistry.

[12] Liu D., Ren D., Huang H., Dankberg J., Rosenfeld R., Cocco M.J., Li L., Brems D.N., Remmele R.L. (2008). Structure and stability changes of human IgG1 Fc as a consequence of methionine oxidation. Biochemistry.

[13] Boyd D., Kaschak T., Yan B. (2011). HIC resolution of an IgG1 with an oxidized Trp in a complementarity determining region. J. Chromatogr. B.

[14] Lu H.S., Fausset P.R., Narhi L.O., Horan T., Shinagawa K., Shimamoto G., Boone T.C. (1999). Chemical modification and site-directed mutagenesis of methionine residues in recombinant human granulocyte colony-stimulating factor: Effect on stability and biological activity. Arch. Biochem. Biophys..

[15] Wang W., Vlasak J., Li Y., Pristatsky P., Fang Y., Pittman T., Roman J., Wang Y., Prueksaritanont T., Ionescu R. (2011). Impact of methionine oxidation in human IgG1 Fc on serum half-life of monoclonal antibodies. Mol. Immunol..

[16] Nguyen T.H., Burnier J., Meng W. (1993). The kinetics of relaxin oxidation by hydrogen peroxide. Pharm. Res..

[17] Hensel M., Steurer R., Fichtl J., Elger C., Wedekind F., Petzold A., Schlothauer T., Molhoj M., Reusch D., Bulau P. (2011). Identification of potential sites for tryptophan oxidation in recombinant antibodies using tert-butylhydroperoxide and quantitative LC-MS. PLoS ONE.

[18] Liu H., Gaza-Bulseco G., Xiang T., Chumsae C. (2008). Structural effect of deglycosylation and methionine oxidation on a recombinant monoclonal antibody. Mol. Immunol..

[19] Niki E. (1990). Free radical initiators as source of water- or lipid-soluble peroxyl radicals. Methods Enzymol..

[20] Werber J., Wang Y.J., Milligan M., Li X., Ji J.A. (2011). Analysis of 2,2′-azobis (2-amidinopropane) dihydrochloride degradation and hydrolysis in aqueous solutions. J. Pharm. Sci..

[21] Steinmann D., Ji J.A., Wang Y.J., Schöneich C. (2012). Oxidation of human growth hormone by oxygen-centered radicals: Formation of Leu-101 hydroperoxide and Tyr-103 oxidation products. Mol. Pharm..

[22] Yoshida Y., Itoh N., Saito Y., Hayakawa M., Niki E. (2004). Application of water-soluble radical initiator, 2,2′-azobis[2-(2-imidazolin-2-yl)propane] dihydrochloride, to a study of oxidative stress. Free Radic. Res..

[23] Sluzky V., Tamada J.A., Klibanov A.M., Langer R. (1991). Kinetics of insulin aggregation in aqueous solutions upon agitation in the presence of hydrophobic surfaces. Proc. Natl. Acad. Sci. USA.

[24] Carpenter J.F., Kendrick B.S., Chang B.S., Manning M.C., Randolph T.W. (1999). Inhibition of stress-induced aggregation of protein therapeutics. Methods Enzymol..

[25] Ratanji K.D., Derrick J.P., Dearman R.J., Kimber I. (2014). Immunogenicity of therapeutic proteins: Influence of aggregation. J. Immunotoxicol..

[26] Finley E.L., Dillon J., Crouch R.K., Schey K.L. (1998). Identification of tryptophan oxidation products in bovine alpha-crystallin. Protein Sci..

[27] Kerwin B.A. (2008). Polysorbates 20 and 80 used in the formulation of protein biotherapeutics: Structure and degradation pathways. J. Pharm. Sci..

[28] Zhao F., Ghezzo-Schöneich E., Aced G.I., Hong J., Milby T., Schöneich C. (1997). Metal-catalyzed oxidation of histidine in human growth hormone. Mechanism, isotope effects, and inhibition by a mild denaturing alcohol. J. Biol. Chem..

[29] Matxain J.M., Padro D., Ristilä M., Strid Å., Eriksson L.A. (2009). Evidence of High •OH Radical Quenching Efficiency by Vitamin B6. J. Phys. Chem. B.

[30] Liu J.L., Lu K.V., Eris T., Katta V., Westcott K.R., Narhi L.O., Lu H.S. (1998). In vitro methionine oxidation of recombinant human leptin. Pharm. Res..

[31] Chu J.W., Yin J., Brooks B.R., Wang D.I.C., Ricci M.S., Brems D.N., Trout B.L. (2004). A comprehensive picture of non-site specific oxidation of methionine residues by peroxides in protein pharmaceuticals. J. Pharm. Sci..

[32] Simat T.J., Steinhart H. (1998). Oxidation of free tryptophan and tryptophan residues in peptides and proteins. J. Agric. Food. Chem..

[33] Mozziconacci O., Arora J., Toth R.T., Joshi S.B., Zhou S., Volkin D.B., Schöneich C. (2016). Site-specific hydrolysis reaction C-terminal of methionine in Met-His during metal-catalyzed oxidation of IgG-1. Mol. Pharm..

[34] Qi P., Volkin D.B., Zhao H., Nedved M.L., Hughes R., Bass R., Yi S.C., Panek M.E., Wang D., Dalmonte P. (2009). Characterization of the photodegradation of a human IgG1 monoclonal antibody formulated as a high-concentration liquid dosage form. J. Pharm. Sci..

[35] Pigault C., Gerard D. (1984). Influence of the location of tryptophan residues in proteins on their photosensitivity. Photochem. Photobiol..

[36] Bommana R., Chai Q., Schöneich C., Weiss W.F., Majumdar R. (2018). Understanding the increased aggregation propensity of a light-exposed IgG1 monoclonal antibody using hydrogen exchange mass spectrometry, biophysical characterization, and structural analysis. J. Pharm. Sci..

[37] Lu D., Liu Z. (2008). Dynamic redox environment-intensified disulfide bond shuffling for protein refolding in vitro: Molecular simulation and experimental validation. J. Phys. Chem. B.

[38] DiMarco T., Giulivi C. (2007). Current analytical methods for the detection of dityrosine, a biomarker of oxidative stress, in biological samples. Mass Spectrom. Rev..

[39] Wang Y., Mattice W.L. (1992). Intramolecular vs intermolecular formation of bityrosine upon photoreaction of poly(l-tyrosine) in dilute aqueous solution. Polym. Bull..

[40] Grewal P., Mallaney M., Lau K., Sreedhara A. (2014). Screening methods to identify indole derivatives that protect against reactive oxygen species induced tryptophan oxidation in proteins. Mol. Pharm..

[41] Yang Y., Strahan A., Li C., Shen A., Liu H., Ouyang J., Katta V., Francissen K., Zhang B. (2010). Detecting low level sequence variants in recombinant monoclonal antibodies. MAbs.

[42] Ren D., Zhang J., Pritchett R., Liu H., Kyauk J., Luo J., Amanullah A. (2011). Detection and identification of a serine to arginine sequence variant in a therapeutic monoclonal antibody. J. Chromatogr. B.

